# Microbiome analysis revealed distinct microbial communities occupying different sized nodules in field-grown peanut

**DOI:** 10.3389/fmicb.2023.1075575

**Published:** 2023-03-02

**Authors:** Md Shakhawat Hossain, Paul B. DeLaune, Terry J. Gentry

**Affiliations:** ^1^Department of Soil and Crop Sciences, Texas A&M University, College Station, TX, United States; ^2^Texas A&M AgriLife Research, College Station, TX, United States; ^3^Texas A&M AgriLife Research, Vernon, TX, United States

**Keywords:** microbiome, metabarcoding, peanut, root nodule symbiosis, endophytes, soil-microbes

## Abstract

Legume nodulation is the powerhouse of biological nitrogen fixation (BNF) where host-specific rhizobia dominate the nodule microbiome. However, other rhizobial or non-rhizobial inhabitants can also colonize legume nodules, and it is unclear how these bacteria interact, compete, or combinedly function in the nodule microbiome. Under such context, to test this hypothesis, we conducted 16S-rRNA based nodule microbiome sequencing to characterize microbial communities in two distinct sized nodules from field-grown peanuts inoculated with a commercial inoculum. We found that microbial communities diverged drastically in the two types of peanut nodules (big and small). Core microbial analysis revealed that the big nodules were inhabited by *Bradyrhizobium*, which dominated composition (>99%) throughout the plant life cycle. Surprisingly, we observed that in addition to *Bradyrhizobium*, the small nodules harbored a diverse set of bacteria (~31%) that were not present in big nodules. Notably, these initially less dominant bacteria gradually dominated in small nodules during the later plant growth phases, which suggested that native microbial communities competed with the commercial inoculum in the small nodules only. Conversely, negligible or no competition was observed in the big nodules. Based on the prediction of KEGG pathway analysis for N and P cycling genes and the presence of diverse genera in the small nodules, we foresee great potential of future studies of these microbial communities which may be crucial for peanut growth and development and/or protecting host plants from various biotic and abiotic stresses.

## Introduction

Bulk and rhizosphere soil contain an array of microbes including bacteria, fungi, algae, protozoa, and archaea (Msimbira and Smith, 2020) with great influence on plant health and nutrient cycling. However, the proper functioning of soil microbial communities depends on many factors including the availability of nutrients, temperature, water, soil structure, pH, and host genotypes and their secreted root exudates (Zgadzaj et al., 2016; Li et al., 2017; Pramanik et al., 2020; Vaidya and Stinchcombe, 2020). An understanding of endophytic or epiphytic soil-microbe interactions with host roots is of great importance because of their potential to promote plant growth in various ways through enhanced nutrient, mineral and water use efficiency, increased production of phytohormones, induced biotic and abiotic stress tolerance, and minimized toxicity of contaminants (Bacon and White, 2016; Kumar et al., 2017; Harman and Uphoff, 2019; Bhattacharyya and Furtak, 2022).

Studies have shown that nitrogen (N_2_) fixation has been a great interest for decades because of the advantage of mutualistic soil rhizobia interactions with the host legumes, thereby developing a symbiotic organ “nodule” (Oldroyd, 2013; Liu and Murray, 2016; Zipfel and Oldroyd, 2017; Lace and Ott, 2018; Poole et al., 2018). Rhizobial bacteria housed in this “nodule” fix atmospheric N_2_ into ammonia (Martínez-Hidalgo and Hirsch, 2017), a source of N for plant growth and development. The nodule formation and development process in legumes is quite complex and highly restricted due to host specificity (Dazzo and Gardiol, 1984; Menéndez et al., 2019; Walker et al., 2020). Unlike the symbiotic host specificity of the nodulation process, a variety of non-rhizobial endophytic or epiphytic strains can mutually interact with both legumes and non-legume crops (Chen et al., 2003; Berg, 2009; Aserse et al., 2013; Martínez-Hidalgo and Hirsch, 2017; Harman and Uphoff, 2019; Olanrewaju and Babalola, 2019; Fadiji et al., 2020; Swarnalakshmi et al., 2020). Some recent microbiome studies have identified that both rhizobial and non-rhizobial strains can eventually nodulate legumes in which the previously known legume nodulation concept of host specificity/restriction for rhizobial interactions changed dramatically (Moulin et al., 2001; Sy et al., 2001; Martínez-Hidalgo and Hirsch, 2017; Mayhood and Mirza, 2021; Bender et al., 2022).

Like other legumes, peanuts can form N_2_-fixing root nodules interacting with host-specific rhizobia in cultivated soils. In general, most legume genera (~ 75%) utilize root-hair dependent infection processes (Quilbé et al., 2022); however, the rhizobial infection process in peanut along with some other legumes (such as *Sesbania*, *Aeschynomene*, *Mimosa*, etc.) is unique and occurs through a “crack entry” mechanism (Boogerd and van Rossum, 1997; Held et al., 2010; Sharma et al., 2020), where rhizobia enter through epidermal cell layer of root tissues at the base of emerging lateral roots and proliferation later occurs into the root cortical cells, subsequently developing nodule primordia and nodules (Bogino et al., 2011; Nievas et al., 2012). Previously published research reported that nodule formation in peanut can occur with a broad range of *Bradyrhizobium* and other rhizobial strains, isolated from different groups of legume plants (Tatiana et al., 2003; Taurian et al., 2006; El-Akhal et al., 2008; Yang and Zhou, 2008; Zaiya Zazou et al., 2018). However, nodulation and N_2_ fixation in peanut can vary depending on numerous factors, such as strains and inoculation practices, geographical locations, soil structure, temperature, water availability, soil acidity, salinity, intercropping, crop rotation, fertilization regime, application of herbicide and pesticide uses, etc. (Rowland et al., 2015).

In addition to the above-mentioned factors, it has been reported that nodule size and root zone depth under the soil (particularly during stress condition) have a direct impact on nodule N_2_ fixation, effectiveness, and activity (Hardarson et al., 1989; King and Purcell, 2001; Voisin, 2003; Tajima et al., 2007). Tajima et al. (2007) found that N_2_-fixing activity directly correlates with the nodule size. For example, medium size nodules (1.5–2.0 mm in diameter) had the highest N_2_-fixing activity per unit fresh weight of nodule compared with the smaller (<1.5 mm) or larger (>2.0 mm) size nodules. In general, nodule formation in legumes is variable and their N_2_-fixing activity may change with developmental stages (Tajima et al., 2007). During early growth stages when nodules are smaller in size, typically, the nodule interior color is white and N_2_-fixing activity is low due to the bacterial cell growth and development. However, at the flowering stage when the nodule color is red/pink and medium in size, N_2_-fixing activity is higher due to active enzyme activity and proper bacterial cell growth. At the late stages of plant growth, although the nodule size may be larger, the nodule color changes to greenish and N_2_-fixing activity rapidly decreases. In addition to the nodule size, effectiveness, and N_2_-fixing activity, the lack of resource (C-source) allocation/or sanctioning by plants to the strains that colonize the nodule can also impact nodule size, development, N_2_ fixation activity due to bacterial competition, and/or fitness (Westhoek et al., 2021).

In previous studies (Ellman-Stortz, 2021), we noticed that peanut roots in experiments at AgriLife research station (Vernon, Texas, United States) formed two types of nodules: regular size N_2_-fixing nodules (hereafter referred to as big nodules) as well as many small nodules in the same plant root system. Interestingly, the growth of these small nodules was arrested yet the nodule development persisted at all growth stages throughout the plant life cycle. Hence, our main objective and research interest was to understand what microbial communities inhabit these small nodules, which might provide a new direction of research opportunities for peanut nodule function and crop improvement.

## Materials and methods

### Field and experimental conditions

The study was conducted under furrow irrigation at the Texas A&M AgriLife Research and Extension Center at Vernon. The soil is classified as a Miles fine sandy loam (Fine-loamy, mixed, super active, thermic Typic Paleustalfs). Peanuts (Span17) were planted on 14 May 2021 at 16 seed m^−1^ using a four-row vacuum planter (1-m row spacing). The preceding year’s crop was cotton. Seed was treated with a commercial inoculum (Exceed^®^ Peat for peanut/cowpea/lespedeza/mung bean; Visjon Biologics) prior to planting. Plots were arranged in a randomized complete block design and replicated four times. Treatments included no cover crop and various cover crop treatments. For this study, peanut plants were collected from the no cover crop control plots only.

### Sample harvesting, surface sterilization, and DNA extraction

Nodules of two independent plant roots were collected from randomized plots in three developmental stages (R2, R4 and R7; Prasad, 1999). Whole peanut plants with roots and nodules were transported overnight on ice from the field site to the laboratory. Roots and nodules were washed thoroughly with tap water and wiped with a paper towel before collecting two types of nodules (big and small) from roots for surface sterilization. Ten big nodules (~ 1.5–2.0 mm), five from each plant, and 20 small nodules (<1.5 mm), 10 from each plant, were selected and immersed in 70% (v/v) ethanol for 2 min, washed with sterile H_2_O three times, and then soaked in 10% (v/v) commercial bleach for 5 min. After bleach treatment, nodules were carefully washed 5–6 times with sterile H_2_O and rinsed of any trace elements of bleach. A total of 12 big and 12 small nodule samples (3 growth stages x 4 plots) were independently collected for total DNA isolation.

Total DNA was extracted from the nodules using DNeasy Plant Pro Kit (Qiagen), according to the manufacturer’s instructions. DNA quality and concentration were determined using a NanoDrop 2000 spectrophotometer (Thermo Scientific, Waltham, United States).

### Amplification of *nifH*, *nodC*, and 16S rRNA genes

To confirm DNA amplifiability, the 16S rRNA gene was amplified using primers *Eub338* and *Eub518* (Fierer et al., 2005). To check for the presence of symbiotic marker genes (*nifH* and *nodC*) in the isolated nodules DNA, primers for *nifH* (Poly et al., 2001) and *nodC* genes (Sarita et al., 2005) were used for PCR amplification. The same PCR conditions were used for all three amplicons (*16S*, *nifH*, and *nodC*) which is as follows: 1 cycle of 95°C for 5 min (preheating), 30 cycles of 95°C for 30 s, 55°C for 30 s, and 72°C for 1 min, and a final extension period at 72°C for 5 min. The size of the PCR product was confirmed by electrophoresis on a 2% agarose gel. For PCR reactions, nuclease-free PCR grade water, 2x GoTaq Master mix, and polymerase were purchased from Promega.

### Library preparation and sequencing

DNA sequencing was performed at the Texas A&M University Institute for Genome Sciences and Society (TIGSS) using Illumina MiSeq. Amplicon of V3-V4 regions of 16S rRNA gene amplified from 10 ng of DNA and 250 bp paired-end library cleaning, indexing, library quantification, normalization, pooling, and loading for MiSeq sequencing was carried out following the standard library preparation guide (Illumina, United States).

### Data analysis of 16S amplicons

Nodule microbiome sequencing results were analyzed with the QIIME 2 software package version 2021.8^1^ (Bolyen et al., 2019) and plugins associated with this version. Raw paired-end sequencing reads were demultiplexed using the demux plugin^2^ with the demux emp-paired command. Quality control, filtering chimeric sequences, and feature table construction were done using the q2-dada2 plugin (Callahan et al., 2016) with trimming parameters (--p-trim-left-f 10 --p-trim-left-r 10 --p-trunc-len-f 250 --p-trunc-len-r 250) based on the demux visualization (QIIME 2 View). Taxonomy classification alignment with 99% similarity was done against the SILVA 138 database (Quast et al., 2012; Yilmaz et al., 2014) using a pretrained naive Bayes classifier and the “feature-classifier” plugin (Bokulich et al., 2018) with the “classify-sklearn.”

Based on “feature table” visualization, an additional filtering step was used to filter out samples with a total feature count less than 3,281. Furthermore, “low abundance features” (i.e., ASV’s with low frequency) were filtered from “feature-table” using “feature-table filter-features” command with “-p-min-frequency 10.” In addition, taxonomy-based filtering was conducted to remove mitochondria, chloroplast, Archaea, and Eukaryota using “taxa-filter-table and taxa-filter-seqs” commands from both taxonomic and rep-seqs tables.

After analyzing raw sequence data, we found more than 5 million sequence reads for both forward and reverse fragments from the 24 nodule microbiome samples with an average count of 111,646 reads per sample generated (Supplementary Table 1). The demultiplexed sequence data resulted an average of 59,560 non-chimeric sequence reads (52.66%; Supplementary Table 2) and inferred a total of 950 amplified sequence variants (ASVs) with an average number of features 40 and mean frequency 59,812 per sample. Since one of the samples (05-PNMB-104R4; Supplementary Table 2) generated a smaller number of non-chimeric sequence reads (6.71%), we further filtered out ASVs based on sampling depth to those that were less than 3,281 feature count/samples, and the minimum feature frequency was set to 10, leaving behind 492 ASVs from 23 samples (lowest feature count was retained to 35,401), which was analyzed further for community diversity and taxonomic classification.

A taxonomic bar-plot was generated using the filtered table and assigned taxonomy file and visualized through the QIIME 2 view website. The resulting filtered sequences from above were used to construct a phylogenetic tree using “align-to-tree-mafft-fasttree” pipeline from the q2-phylogeny plugin.^3^ Subsequently, phylogenetic diversity metrics were constructed with the resulting output “rooted phylogenetic tree.” Alpha and beta diversities were calculated through the q2-diversity plugin^4^ using the “core-metrics-phylogenetic” method with a sampling depth (rarefaction) of 1,290. Associations between categorical metadata columns (two types of nodule samples) and alpha diversity data were calculated through “qiime diversity alpha-group-significance” with Kruskal-Wallis pairwise test (Kruskal and Wallis, 1952) for Shannon’s diversity index. In addition, we also used PERMANOVA (default) to evaluate if the distances between the two nodule groups (sample compositions) were similar using the “qiime diversity beta-group-significance” plugin (Anderson, 2001) for weighted UniFrac distance. Further, we also conducted Mann–Whitney/Wilcoxon significance tests for alpha and beta diversity in two nodule groups and Kruskal-Wallis pairwise test for beta diversity analysis in three developmental growth stages of small nodules using the “rstatix” package in R. To find similarity within samples or dissimilarity between sample groups, a principal component analysis (PCA) was conducted using the centered log ratio (CLR) transformed microbial compositions. The PERMANOVA (*n* = 999) was done on Aitchison distance matrices to test significant effect of microbial compositions between two nodule types using the vegan package in R. A compositional heatmap of CLR-transformed microbial abundance (prevalence = 5) across samples was made with R package “microViz.”

To identify differentially abundant core microbial communities from two types of nodule samples, we used a statistical power analysis called “ANCOM” (analysis of composition of microbiomes) using ANCOM plugin (Mandal et al., 2015) with “qiime composition ancom” command. For ANCOM analysis, the ASV table that filtered out for low features, frequency, and for chloroplast, mitochondria, Archaea, and Eukaryota was used. Initially, taxa were collapsed using “qiime taxa collapse” command with - -p-level 6 to create a genus level table and later, add-pseudocount 1 for log0 (if any), and finally, “qiime composition ancom” command was used to tabulate significant (W-score) groups of bacteria at genus level that were differentially abundant (Estaki et al., 2020).

To predict functional profiles of pathways from 16S rRNA gene sequences of big and small nodule microbiome data (i.e., ASVs and abundance), we analyzed functional metabolic pathways for KO, EC, TIGRFAM, COG, CAZymes, and Pfam using a recently published metagenome analysis tool called “MicFunPred” (Mongad et al., 2021).

All the bioinformatics and biostatistics data were analyzed using Qiime-2 (Bolyen et al., 2019), Phyloseq (McMurdie and Holmes, 2013), and R packages in R Studio platforms (RStudio Team, 2020), and all the figures were generated using R version 4. 1. 2 (R Core Team, 2013).

### Data availability statement

Raw sequencing data were submitted to the NCBI Sequence Read Archive (SRA) under Bio Project accession PRJNA865795 and will be available upon publication. Data files and scripts that were used to generate figures are available at github.com/shak71/PNM.

## Results

### Peanut forms a distinct pattern of nodulation in the field

**Figure 1:**
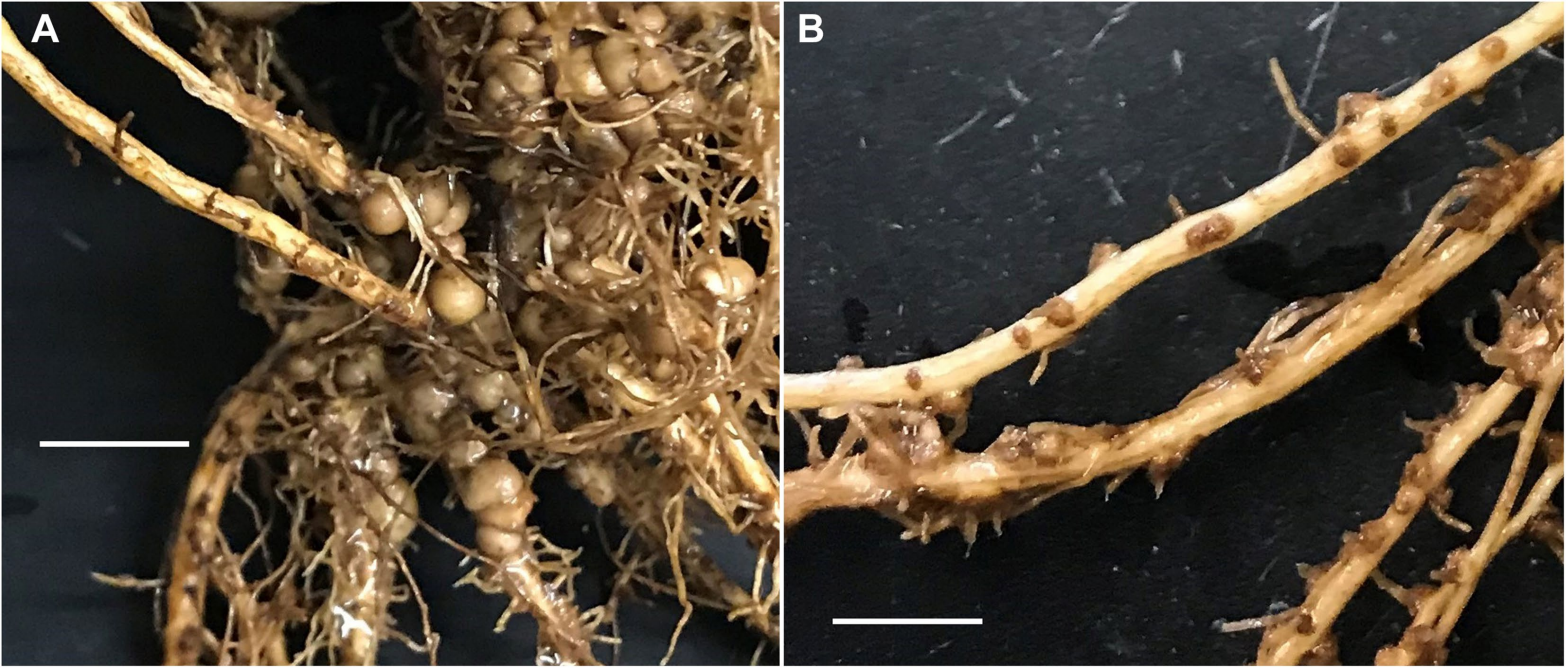
Field-grown peanut root-nodules used as a source of DNA for 16S-based microbiome sequencing and analysis. **(A,B)** represent big nodules and small nodules, respectively. The images were taken with the same magnification (scale 50  mm).

Despite being inoculated with host-specific rhizobia, the field-grown peanuts formed both regular (referred to as big) sized (Figure 1A) and small nodules (Figure 1B; Please see M&M section for nodule size). These small nodules were present at all observed growth stages throughout the plant life cycle. While the larger nodules had a red interior color suggesting active N_2_ fixation, the small nodules showed variable color (white, light pink, and gray or green) suggesting limited N_2_ fixation activity (data not shown). However, N_2_ fixation genes were found in both types of nodules. The *nifH* gene was found in all big and small nodules, except one small nodule while the *nodC* gene was not amplified from few small nodule samples (Supplementary Table 3).

### Diversity analysis of bacterial communities in peanut nodules

**Figure 2:**
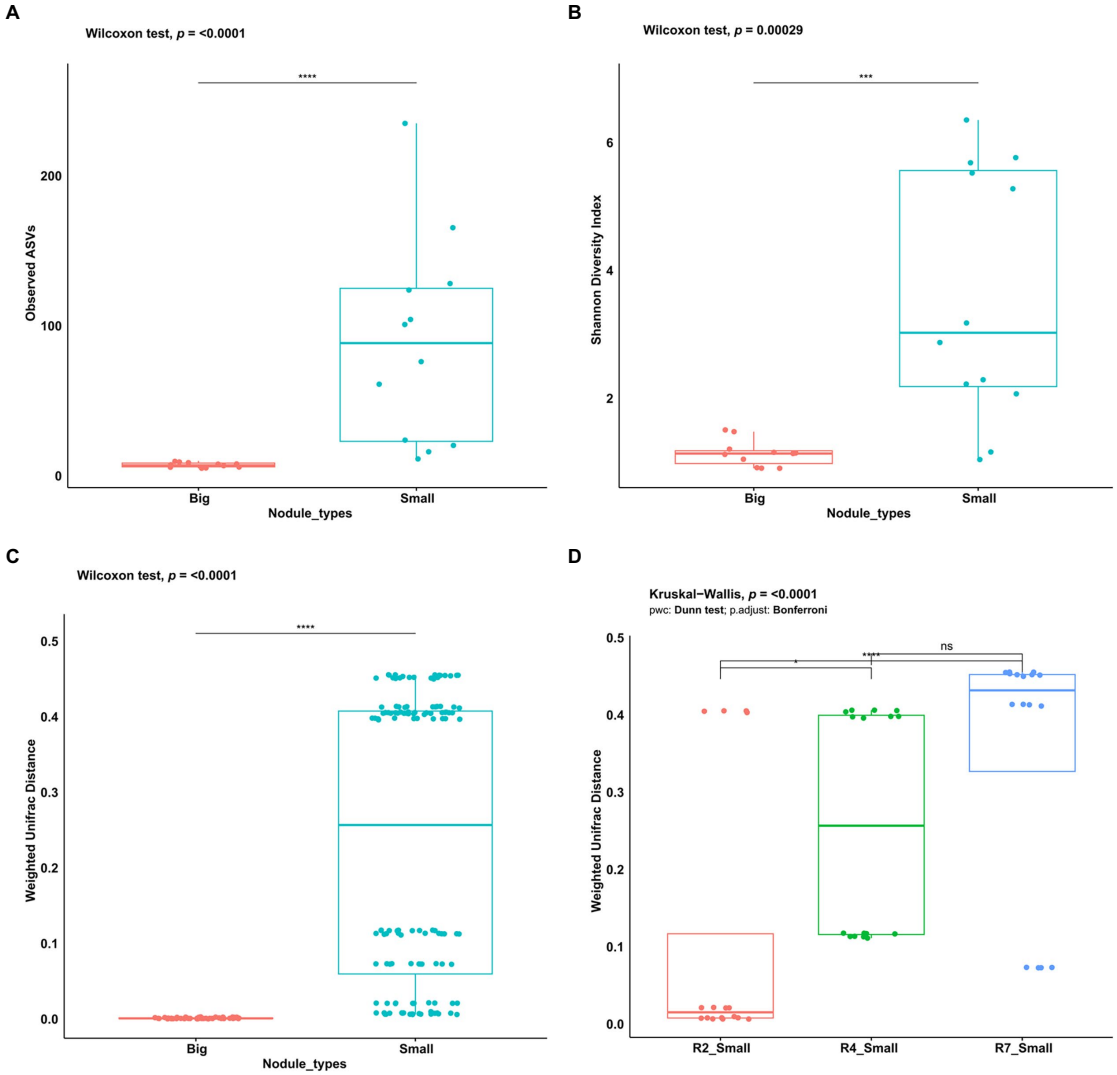
Alpha and beta diversity indices of peanut big and small nodule microbiome (16S rRNA gene). **(A)** Number of observed ASVs, **(B)** Shannon diversity index, **(C)** Weighted UniFrac distance for nodule types, and **(D)** three developmental growth stages of small nodules. For significance analysis, Mann–Whitney/Wilcoxon tests were used for observed ASVs, Shannon diversity index, Weighted UniFrac distance. Kruskal-Wallis test with pairwise comparison was used for Weighted UniFrac distance in three small nodules developmental growth stages. *p* values are denoted with an * *p* ≤ 0.05; **, *p* ≤ 0.01; ***, *p* ≤ 0.001; ****, *p* ≤ 0.0001 and ‘ns’ indicate no significance. Dots denote number of ASVs, respective alpha and beta diversity estimate values of nodule samples grouped based on their size and three developmental growth stages of small nodules.

To assess and investigate the bacterial community richness and diversity in two nodule types, the alpha diversity measures for observed ASVs and Shannon Diversity Index were calculated in big and small nodules of peanut roots. The number of observed ASVs differed significantly (*p* = <0.0001) between the two nodule types (Figure 2A). Observed ASVs in big nodule samples ranged from 5 to 10 while in small nodule samples ranged from 11 to 235. A Mann–Whitney/Wilcoxon significance test analysis for Shannon Diversity index (Figure 2B) was used to find the community richness which indicated a significant difference and variation between the big and small nodule microbiomes (*p* = 0.00029) with the bacterial communities in the small nodules being more diverse than those in the big nodules.

**Figure 3:**
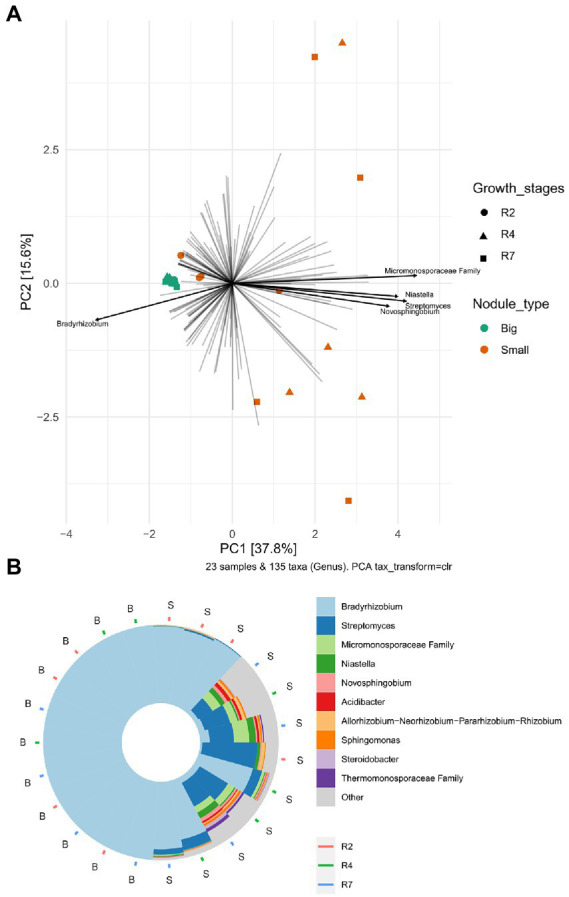
Principal Component Analysis (PCA) and sample compositions of bacterial communities in big and small nodules of peanut roots. **(A)** Centered log-ratio transformed (CLR) an unconstrained genus level PCA analysis in peanuts big (11) and small (12) nodule samples and three developmental growth stages (R2, R4, R7). The top five taxa (labeled with their annotations) were indicated by bold black lines. **(B)** Iris plot showing the sample compositions of microbiota at the genus level in big and small nodule growth stages (R2, R4, and R7). The big (B) and small (S) nodule samples on the iris plot were automatically arranged by their rotational position around the center/origin of the PCA plot. Both PCA and iris plots were performed with R package “microViz.”

To analyze the bacterial community compositions in two types of nodules as well as growth stages for small nodules, the beta diversity index was calculated based on Weighted UniFrac distances. A Mann–Whitney/Wilcoxon significance test analysis was used among all samples grouped as big and small for Weighted UniFrac distance for the significance test and the results clearly showed that bacterial compositions differ between big and small nodule types (*p* = < 0.0001; Figure 2C). Since bacterial compositions differ within the small nodule category, we further explored the possibility within three developmental growth stages of small nodules and indeed, we found that bacterial compositions differ significantly (Kruskal-Wallis test, *p* = <0.0001) among all three growth stages of small nodules (Figure 2D). Further compositional aspects of centered log ratio (CLR) transformed PCA were analyzed for the bacterial communities in the big and small nodules as well as three different growth stages (R2, R4, and R7) of the small nodules. Based on the PERMANOVA test, the PCA results indicated that there were significant differences in bacterial communities between big and small nodules (*p* = 0.001); however, the bacterial communities among three growth stages of small nodules were also slightly different (*p* = 0.061; Figure 3A). Within small nodules, bacterial communities tended to group separately in these three growth stages except one or two data points in R4 and R7 stages that were closer to other growth stages. In contrast, bacterial communities in the big nodules seemed to cluster together and vary less across growth stages. Notably, the small nodule communities were initially (R2) somewhat similar to the big nodules but became more distinct at later growth stages (R4 & R7). At the genus level, based on top taxa loading on the PCA plot as well as direct visualization of sample compositions on the iris plot (Figures 3A,B), big nodule samples contained mostly *Bradyrhizobium*, while other taxa were often more abundant in small nodule samples. Collectively, the data clearly indicated that the small nodules had more diverse and temporally variable bacterial communities than the big nodules.

### Bacterial community composition in big and small nodules of peanut roots

**Figure 4:**
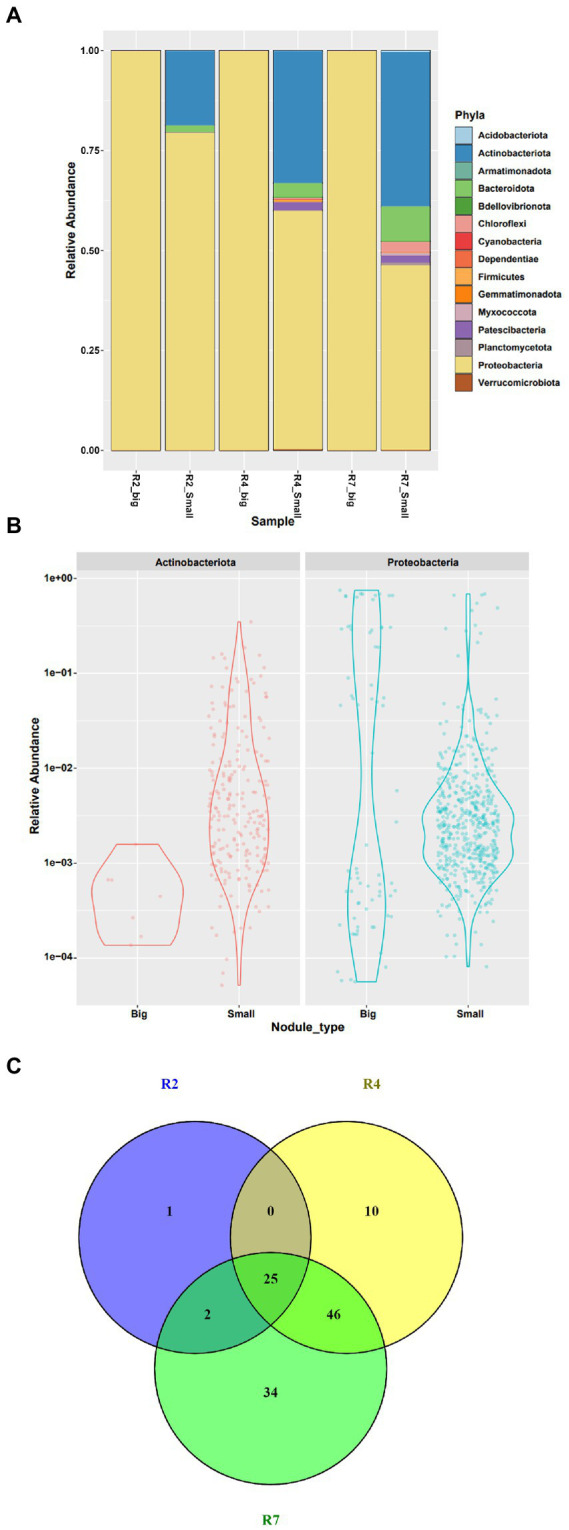
Relative abundance of bacterial communities at phylum level in **(A)** big and small nodules of peanut roots, **(B)** distribution and relative abundance of microbial communities of two major phyla Actinobacteriota and Proteobacteria, and **(C)** a Venn diagram showing the taxa at genus level from three developmental growth stages of small nodule type. For Venn diagram, taxa were selected based on relative abundance >0 for three growth stages of small nodules (R2, R4 and R7), and was made using Venny 2.1.0 (https://bioinfogp.cnb.csic.es/tools/venny/).

All the filtered ASV’s from the big and small nodules were classified as bacteria and included 15 phyla, 25 classes, 57 orders, 87 families, and 151 genera (Figure 4; Supplementary Table 4). Both nodule types had large relative populations of Proteobacteria (Figure 4A), with the phylum representing ~99.96% of the bacterial community in the big nodules and ~ 68.67% in the small nodules combinedly from three growth stages. Thus, the big nodules harbored a tiny fraction of native community microbes while the small nodules were inhabited by a large portion (~31.32%) of diverse microbes. Further analysis revealed that only Actinobacteriota (~0.03%) co-inhabited with Proteobacteria in the big nodules; however, 15 different phyla including Actinobacteriota were detected in the small nodules (Figure 4B).

Interestingly, the microbiome data showed that these diverse bacterial populations in the small nodules dominated over time, becoming more abundant at later growth stages – the most noticeable being members of the Actinobacteriota, Bacteroidota, Chloroflexi, and Patescibacteria (Figure 4A). Surprisingly, the phyla Patescibacteria and Chloroflexi were not enriched at all in the early R2 growth stage. In addition, we found that Proteobacteria abundance and occupancy in the big nodules was steady throughout the growth stages. However, Proteobacteria were comparatively lower while the diverse microbial populations appeared higher in the small nodules irrespective of growth stages. At the phylum level, the microbial abundance and distribution frequency was analyzed since the most dominant and abundant phyla were Proteobacteria and Actinobacteriota compared to other taxa. The data revealed that microbial abundance and frequency varied from low to high abundance in the big and small nodules for these two phyla with more distinct bacterial populations present in small nodules. To further explore the variation of microbiota within these three growth stages of the small nodules, 118 taxa at the genus level were extracted based on relative abundance without any uncultured taxa (Figure 4C; Supplementary Table 5). The Venn diagram showed the taxa (relative abundance >0) distribution in R2, R4 and R7 growth stages of small nodules, and indicated there was diverse microbial dominance over the growth cycle of the small nodules. Interestingly, we also found some unique as well as overlapped microbiota between these three growth stages with the greatest overlap between growth stages R4 and R7.

**Figure 5:**
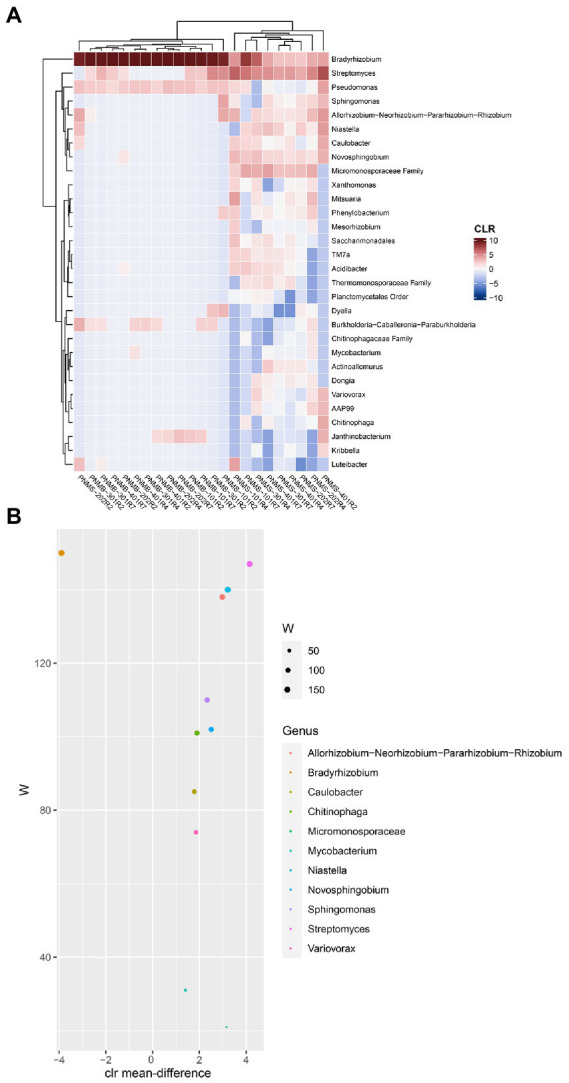
Microbial abundance and the differentially abundant core bacteria in big and small nodules of peanut roots. **(A)** Heatmap showed the centered log-ratio transformed (CLR) microbial abundance at the genus level. The x and y-axis indicate sample name and the microbial taxa, respectively. Peanut nodule microbiome big (PNMB) and small (PNMS) denoted on the x-axis. 101, 202, 301, and 401 represent biological replications, and R2, R4, and R7 represent three developmental growth stages. **(B)** The plot showed the differentially abundant core microbiota at the genus level in big and small nodules. The differentially abundant core microbiota was selected based on ANCOM analysis as stated in the Materials and Methods section. The W value represents the number of times the null hypothesis (the average abundance of a given genus in a group is equal to that in the other group) was rejected for a given genus. The x-axis value represents the CLR-transformed mean difference in abundance of a given genus between the big and small nodule groups. A positive x-axis indicates a genus is abundant in a small nodule group and a negative x-axis value indicates a genus is abundant in a big nodule group. Only genera with W value >20 were plotted (color coded).

To identify the dominant and abundant taxa in the big and small nodules, we first removed all the unclassified species-level data, the rare and uncultured taxa, the taxa with a minimum prevalence of 5, and then a CLR-transformed compositional heatmap was made at the genus level (Figure 5A). The small nodule samples from the three growth stages had a diverse bacterial community while the big nodule samples were dominated by a single genus *Bradyrhizobium* spp. The core bacterial genera in the big and small nodules were identified using ANCOM differential test analysis at the genus level (Figure 5B). Based on ‘W’ score > 100, 7 genera were identified: *Bradyrhizobium*, *Streptomyces*, *Niastella*, *Allorhizobium-Neorhizobium-Pararhizobium-Rhizobium*, *Sphingomonas*, *Novosphingobium*, and *Chitinophaga*. These seven core genera were also found in the compositional heatmap, suggesting that these are the dominant and highly abundant core genera in big and small nodules. Of these seven abundant and dominant core genera, based on W score, only *Bradyrhizobium* was highly abundant in the big nodules, while at least 6 genera including *Streptomyces, Allorhizobium-Neorhizobium-Pararhizobium-Rhizobium, Niastella, Novosphingobium*, *Sphingomonas,* and *Chitinophaga* were primarily abundant in the small nodules.

### Prediction of functional pathway genes of bacterial communities

**Figure 6:**
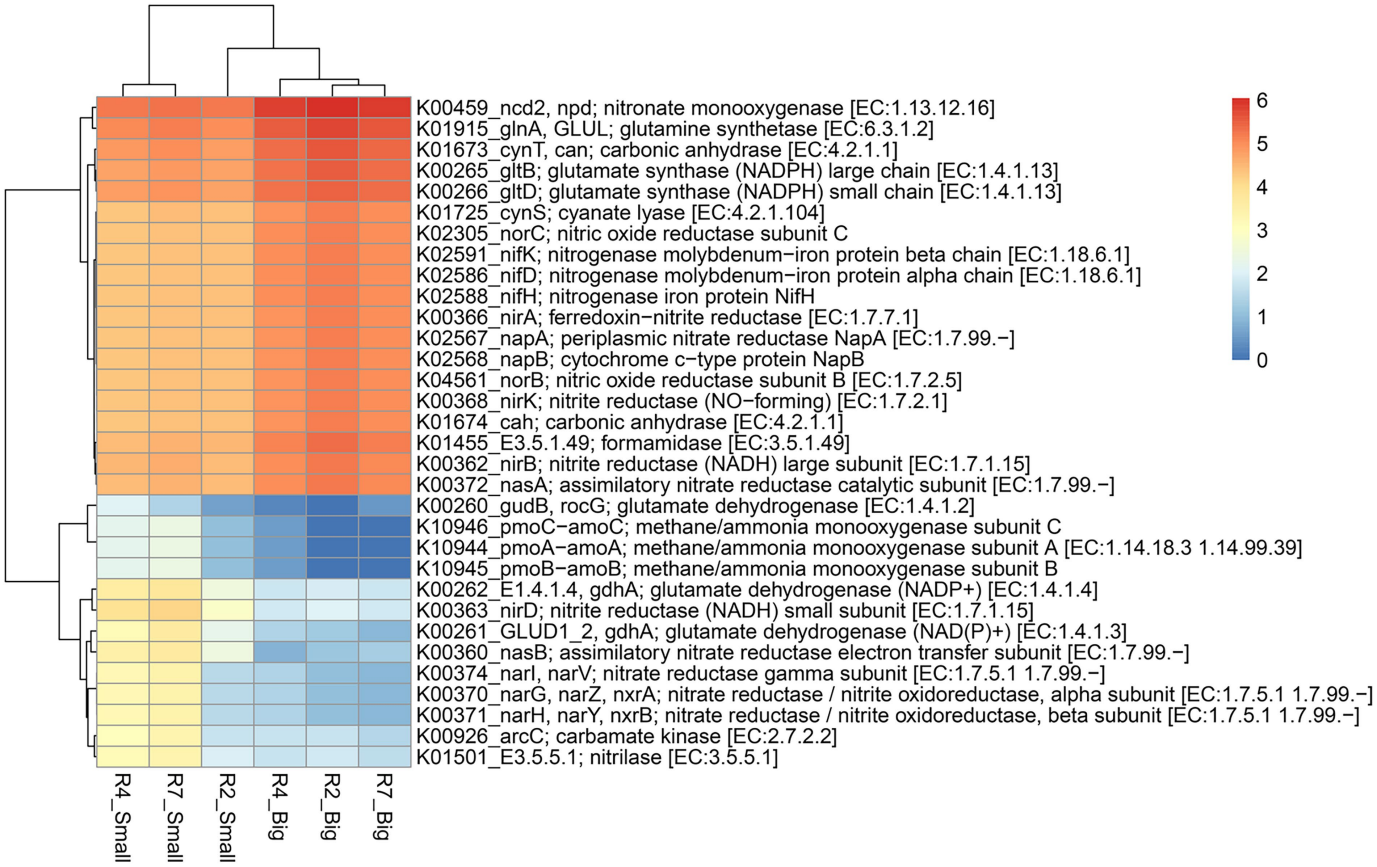
Heatmap of the predicted KEGG Orthology (KO) pathway genes of bacterial communities from three developmental stages of big and small nodules in peanut roots related to N cycling processes. The scale bar indicates the color saturation gradient based on log10 transformed values of pathway abundances enriched from bacterial taxa in big and small nodules. R2, R4, and R7 represent three developmental growth stages. The partial data for genes related to N cycling processes used to plot the heatmap and the full set of enriched pathways data is listed in Supplementary Table 6.

**Figure 7:**
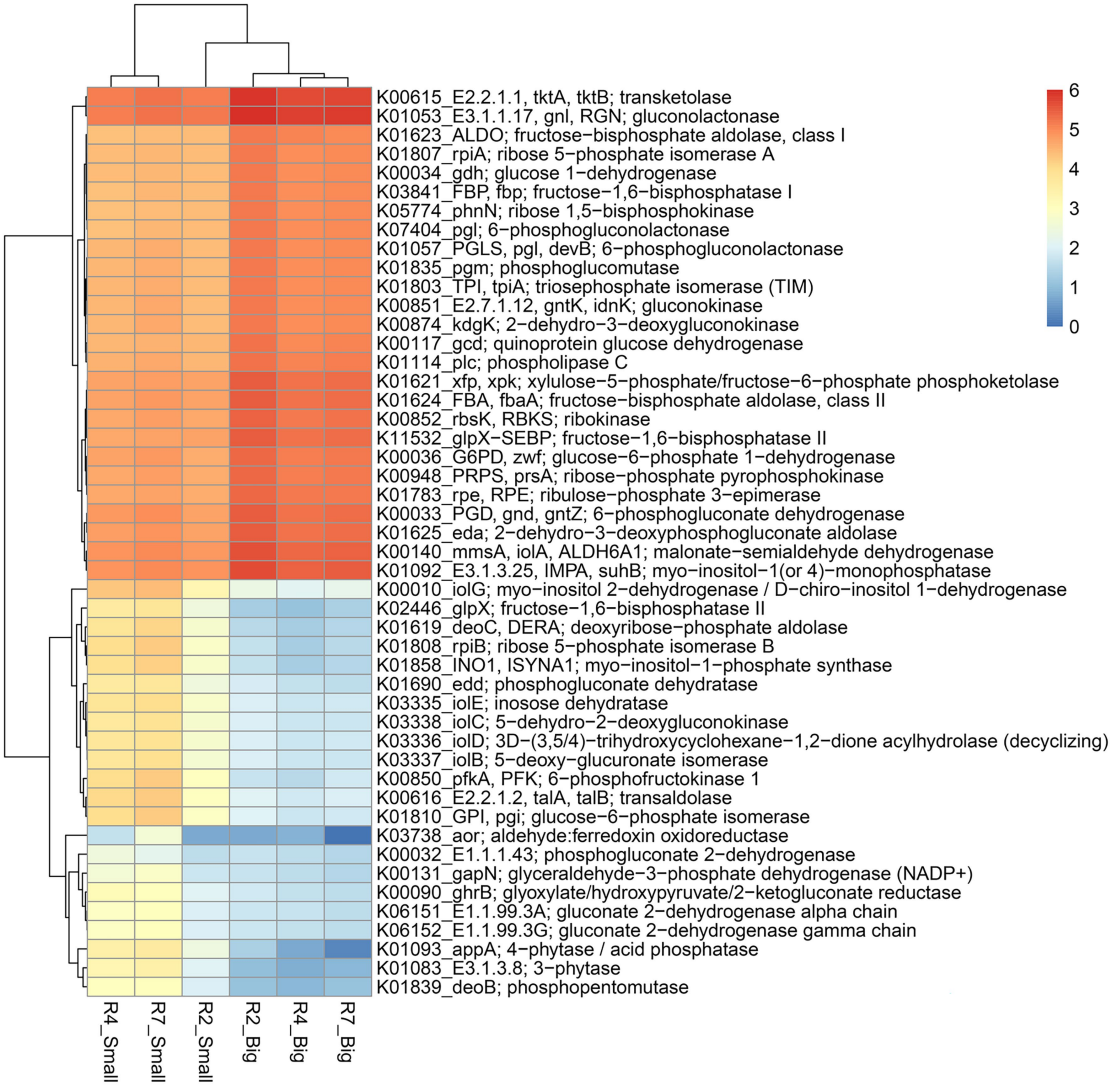
Heatmap of the predicted KEGG Orthology (KO) pathway genes of bacterial communities from three developmental stages of big and small nodules of peanut roots related to P cycling processes. The scale bar indicates the color saturation gradient based on log10 transformed values of pathway abundances enriched from bacterial taxa in peanut big and small nodules. R2, R4, and R7 represent three developmental growth stages. The partial data for genes related to P cycling processes used to plot the heatmap and the full set of enriched pathways data is listed in Supplementary Table 6.

Predicted functional profiles of pathway genes (based on pathway abundance) of bacterial communities from big and small nodules of peanut roots were generated using ‘MicFunPred’ (Mongad et al., 2021). Utilizing 16S rRNA gene sequences and normalized ASV’s abundance from three developmental stages of big and small nodules, metagenomes in terms of KEGG Orthology (KO) functional categories (based on log10 transformed pathways abundance) were used to investigate the enriched function for pathway genes related to nitrogen (N) and phosphorus (P) cycling processes (Figures 6, 7; Supplementary Table 6). Based on search criteria as “nitrogen metabolism” at C-level hierarchy (Supplementary Table 6), a total of 32 genes from the big nodules and 36 genes from the small nodules related to the N cycling process were enriched. A heatmap was made based on all the 32 genes that were common in both big and small nodule developmental stages (Figure 6). The relative abundance of most biological N_2_ fixation (*nifH*, *nifD,* and *nifK*), assimilatory (*nirA*, *nasA,* and *nasB*) and dissimilatory (*nirB*, *napA,* and *napB*) nitrate reduction, and denitrification (*norB* and *norC*)-related genes were higher in all three growth stages of big nodules compared to small nodules at all growth stages (Figure 6). However, the predicted pathway data showed that the relative abundance of some genes or different subunits related to the N cycling process was higher in at least two growth stages (R4 and R7) of the small nodules. For example, *nirD*—*nitrite reductase* (*NADH*) small subunit, *nitrilase*, *gdhA*—*glutamate dehydrogenase* (*NADP+*), *nasB*—assimilatory *nitrate reductase* electron transfer subunit, *GLUD1_2*, *gdhA*—*glutamate dehydrogenase* (*NAD(P)+*), *arc*—*carbamate kinase*, *nitrate reductase* / *nitrite oxidoreductase* alpha subunit (*narG*, *narZ*, *nxrA*), *nitrate reductase* / *nitrite oxidoreductase* beta subunit (*narH*, *narY*, and *nxrB*), and *nitrate reductase* gamma subunit (*narI*, *narV*; Figure 6). Interestingly, a few genes related to N cycling processes that were uniquely enriched only in the small nodules were observed. These unique nitrogen-related pathway genes were *Glutamate synthase* (*ferredoxin*; *GLU*, *gltS*), *ferredoxin-nitrate reductase* (*narB*), *nitrous-oxide reductase* (*nosZ*), and *hydroxylamine reductase* (*hcp*; Supplementary Table 6).

**Figure 8:**
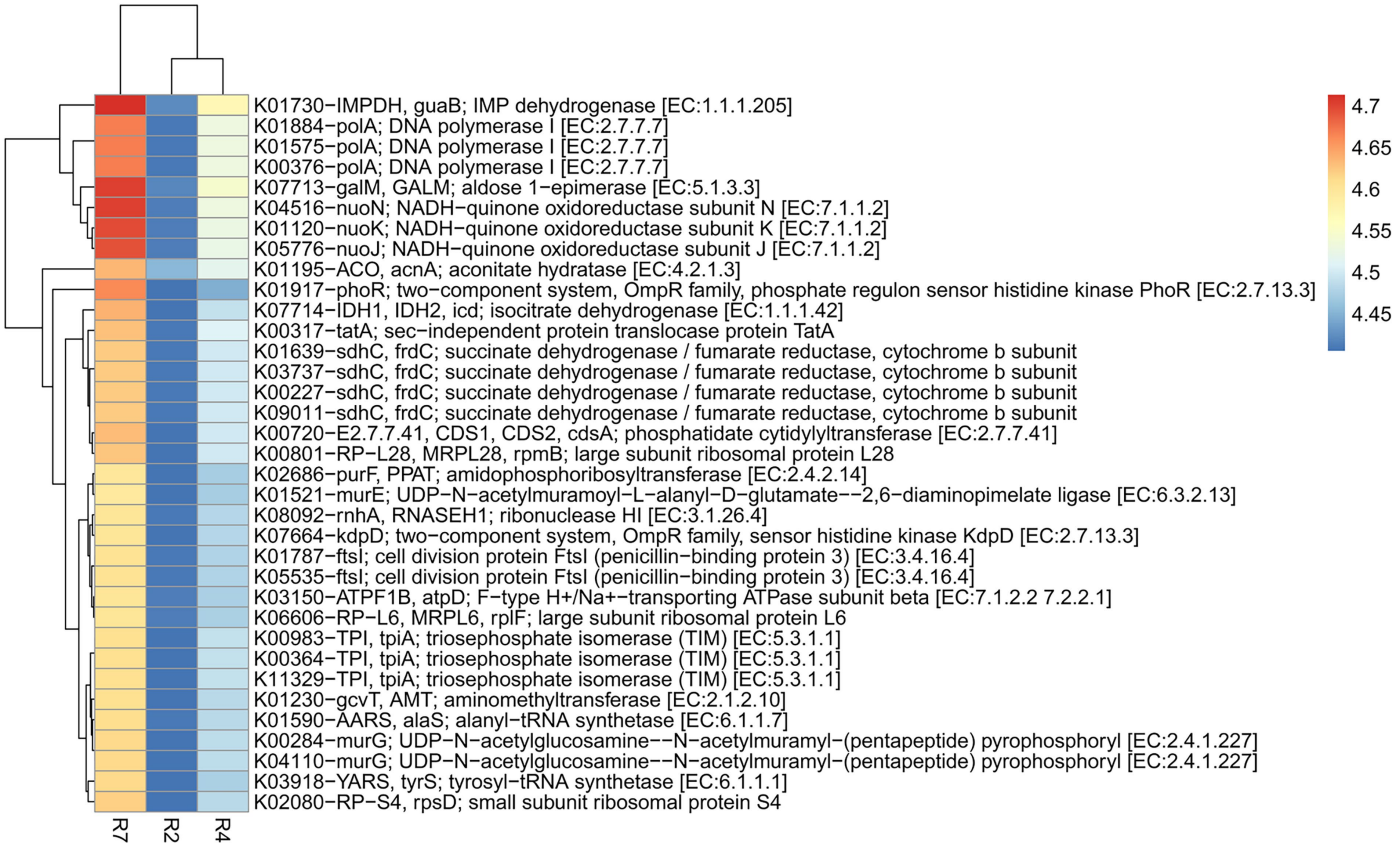
Heatmap of top 35 predicted KEGG Orthology (KO) pathway genes of bacterial communities that were unique to three developmental stages of small nodules of peanut roots. The scale bar indicates the color saturation gradient based on log10 transformed values of pathway abundances enriched from bacterial taxa in small nodules growth stages. R2, R4, and R7 represent three developmental growth stages.

Like the N cycling processes, KO pathway genes related to the P cycling processes were analyzed (Figure 7). Based on search criteria “phosphate metabolism/pathway” at C-level hierarchy (Supplementary Table 6), a total of 48 genes from three growth stages of big nodules and 58 genes from small nodules related to P cycling processes were enriched. A heatmap was made based on all the 48 genes that were common in both big and small nodule developmental stages (Figure 7). Among 48 phosphate-related genes, almost 50% (26) genes showed higher relative abundance in big nodules growth stages compared to small nodules. However, the relative abundance of 22 genes related to P cycling processes was also higher in all three stages of small nodules category, particularly *3-phytase* and *4-phytase*/*acid phosphatase*, *myo-inositol 2-dehydrogenase* (*iolG*), *fructose-1,6-bisphosphatase II* (*glpX*), *deoxyribose-phosphate aldolase* (*deoC*), *ribose 5-phosphate isomerase B* (*rpiB*), *myo-inositol-1-phosphate synthase* (*INO1*), *6-phosphofructokinase 1* (*pfkA*), *transaldolase* (*talA*, *talB*), and *glucose-6-phosphate isomerase* (*GPI*; Figure 7). The unique phosphate-related genes enriched in small nodules were *inositol oxygenase* (*MIOX*), *1-phosphatidylinositol phosphodiesterase* (*plc*), *2-keto-myo-inositol isomerase* (*ioll*), *diphosphate-dependent phosphofructokinase* (*pfp*), *6-phospho-3-hexuloisomerase* (*hxlB*), *3-hexulose-6-phosphate synthase* (*hxlA*), *dehydrogluconokinase* (*kguK*), *glucose-6-phosphate isomerase*-archaeal (*pgi1*), and *CDP-diacylglycerol--inositol 3-phosphatidyltransferase* (*CDIPT*), and *fructose-1,6-bisphosphatase III* (*fbp3*). Besides N & P cycling genes, other pathway genes were analyzed that were unique only in the small nodule growth stages. All the duplicates for KO identifications from both big and small nodules were removed, and then sorted out for unique pathway genes in small nodule category only, of which 383 genes were observed that were unique and enriched (log10 abundance >4.0 in all three stages; Supplementary Table 7). Among the observed 383 pathway genes, a heatmap was made for 35 genes that were common among three stages of pre-selected top 50 pathway genes (Figure 8). All 35 pathway genes were highly enriched in growth stage R7 compared to R2 and R4, suggesting that these genes might play some role in the late stage of the small nodules. The most noticeable and highly enriched genes were *IMP dehydrogenase* (*IMPDH*, *guaB*), *NADH-quinone oxidoreductase subunit J*, *K*, *N* (*nuoJ*, *nuoK*, and *nuoN*), and *succinate dehydrogenase* / *fumarate reductase* (*sdhC*, *frdC*), and *phosphate regulon sensor histidine kinase PhoR* (*phoR*). It is quite interesting to observe that some key metabolic categories other than N and P were specifically increased in the small nodules (Supplementary Table 7) and this warrants future studies for further characterization.

## Discussion

In this study, the nodule microbiome from two distinct types of nodules formed on the same field-grown peanut roots was characterized using a 16S rRNA amplicon strategy. Interestingly, we noticed that the occurrence and abundance of small nodule phenotype was consistent across plant growth stages, as observed in previous studies (Ellman-Stortz, 2021). It is well known that the lack of some host genetic components tightly regulates bacterial infection processes and may control this type of phenotype (Hossain et al., 2012). However, it was observed that the field-grown peanut with the commercial seed coating inoculum formed small nodules and persisted at all growth stages throughout the plant life cycle alongside the regular N_2_-fixing nodulation. There is evidence that small nodules may result from resource sanctioning by the plant in response to specific nodule occupants (Westhoek et al., 2021); however, the microbiome composition of these smaller nodules has not been extensively investigated, especially for peanuts. This was the impetus to understand and classify the inhabitants of these two distinct nodule types.

It was observed that *nifH* and *nodC* (symbiotic marker genes) were generally present in both the big and the small nodules, suggesting that bacterial communities in these nodules might have the ability to fix N_2_. We did not directly measure the gene expression or N_2_-fixing activity in these nodules, but the examination of several nodules indicated a light pink/green color in small nodules and pink color in big nodules (data not shown). Also, previous reports suggest that small nodules (<1.0 mm) with light red/pink color might have less N_2_-fixing activity than medium sized (1.5–2.0 mm) nodules (Tajima et al., 2007). The non-detection of *nifH* or *nodC* gene in a few small nodule samples might be due to the low copies of the gene in the genome or the possibility of missing genes from the bacterial genomes. The latter is less likely because the same samples in other replications and/or growth stages showed the presence of the gene (Supplementary Table 3).

After utilizing filtration criteria, our 16S rRNA analyses from nodules revealed only 492 ASV’s. The big nodules had less observed ASV’s with a higher frequency while small nodules possessed more ASV’s compared to big nodules with comparatively less frequency, which further indicates a tiny fraction of community diversity/occupants in big nodules. Since most of the ASV’s came from small nodules, initial analysis of these ASV’s clearly indicates that small nodule occupants were more diverse than big nodules, which might be the reason for the bimodal distribution patterns. Further analysis of this data through taxonomic community profiling found more diverse bacterial communities occupied in the small nodules compared to the big nodules, suggesting that these diverse microbial communities in the small nodule might play some role(s) for microbe-microbe and/or microbe-plant interactions for the benefit of plant growth promotion, microbe/plant adaptation to harsh environmental stress, and/or protecting from disease or environmental pollution.

It is noteworthy to mention that peanut seeds used for the experiment were coated with commercial inoculum before planting. Since rhizobia inoculants often fail to compete for nodule occupancy against the native soil rhizobia due to the strain’s variation, abundance, and composition as well as many environmental factors (Ji et al., 2017; Mendoza-Suárez et al., 2021), we had the opportunity to look at the possibility of rhizobia competition and occupancy in our data set. In the context of competition and occupancy prospective, we did not see any major evidence for native microbial community members displacing the commercial inoculum in the big nodules, suggesting that commercial inoculum might be active in big nodules throughout the plant growth cycle. However, it was quite surprising to observe that a diverse set of native microbial community members occupied the small nodules. These native communities gradually dominated over time in the small nodule compartments but did not have any clear indication about why native microbial communities colonized small nodules from the early stage and kept competing throughout the peanut life cycle. However, our data from microbial composition analysis as well as core dominant microbial community profiling of two types of nodule categories provide some intriguing insights into this. Based on both microbial composition analysis and core dominant microbial genera observed, only *Bradyrhizobium* was abundant in the big nodules while *Streptomyces* were primarily abundant & dominant in the small nodules, along with several core genera including *Allorhizobium-Neorhizobium-Pararhizobium-Rhizobium*, *Niastella*, *Novosphingobium*, *Sphingomonas, Chitinophaga,* and *Caulobacter*, that solely occupied the small nodules.

Though we could not directly correlate biological and physiological functions of these core bacteria that occupied the small nodules either as beneficial or commensal and/or pathogenic (Brader et al., 2017), some previous reports highlighted the beneficial role and importance of these endophytic bacterial taxa (Aserse et al., 2013; Khan et al., 2014; Rodriguez-Conde et al., 2016; McKee et al., 2019; Olanrewaju and Babalola, 2019; Asaf et al., 2020; Segura et al., 2021). For example, the genus *Niastella*, from the Chitinophagales order under phylum Bacteroidota, can degrade a variety of carbohydrate-based biomass, releasing sugars as nutrients for their own growth as well as for other microbial communities (McKee et al., 2019). Recent studies found that bacterial species from *Novosphingobium*, *Sphingomonas,* and *Niastella* genera play significant roles: from remediation of environmental contamination to producing highly beneficial phytohormones (gibberellins and indole acetic acid, sphingan, and gellan gum), improving plant-growth attributes (i.e., shoot length, chlorophyll contents, and shoot and root dry weight) protecting plants from stress conditions such as drought, salinity, and heavy metals in agriculture soils, co-metabolizing nutrients existing in root exudates, and producing the N-acyl-homoserine lactone quorum-sensing (QS) signals (Gan et al., 2009; Khan et al., 2014; Rodriguez-Conde et al., 2016; Asaf et al., 2017, 2020; Segura et al., 2021).

The genus *Streptomyces* under phylum Actinobacteriota was almost exclusively detected in the small nodules. Most species in this group are widely distributed as soil microbes, are efficient as rhizosphere, rhizoplane, and endophytic colonizers of host plants, and are a useful source for producing bioactive compounds, antibiotics, and extracellular enzymes. Thus, this group of bacteria functions for plant growth promotions as a biocontrol agent as well as a biofertilizer. As biocontrol agents, they synthesize plant growth regulators, siderophore production, antibiotic production, and volatile compound secretion. As biofertilizer, they directly promote plant growth promotion by the production of phytohormones (such as auxins, cytokinins, and gibberellins), the scavenging of ferric iron from the environment by siderophores, N_2_ fixation, and the suppression of stress response in plants by production of *1-aminocyclopropane-1-carboxylate* (*ACC*) *deaminase* activity (for details please see the review Olanrewaju et al., 2019).

Although there are several tools available to predict functional profiles of 16S rRNA gene sequence data (DeSantis et al., 2006; Pruesse et al., 2007; Douglas et al., 2020; Narayan et al., 2020; Wemheuer et al., 2020), a recently developed bioinformatic tool “MicFunPred” (Mongad et al., 2021) was utilized because of its advantages using a novel approach to predict and understand the functional profiles of the metagenome based on a set of core genes only, minimizing false-positive predictions in the microbial communities of big and small nodules of the peanut roots. Here, the KO functional category for genes related to N and P cycling processes were reported. Relative abundance of many genes were observed related to the N and P cycling processes, which were higher in abundance in big nodules compared to small nodules. However, it was quite interesting that a few genes/subunits showed clearly higher abundance in the small nodules compared to the big nodules. For the N cycling process in small nodule microbiota, *nitrate reductase*/*nitrate oxidoreductase* alpha, beta, and gamma subunits (*narG*, *narH*, and *narl*), *carbamate kinase* (*arcC*), and *glutamate dehydrogenase* (*NADP^+^*; *gdhA*; *GLUD1_2*) were high in abundance. *Nitrate reductases* reduce nitrate by microbial dissimilatory processes for denitrification or dissimilatory reduction of nitrate to ammonium. The presence of these subunit/genes with higher abundance in the small nodule microbiota suggest they might participate in the N_2_-fixation process. Another example is that *glutamate dehydrogenase* (*NADP^+^*; *gdhA*; *GLUD1_2*) was higher in the small nodule microbiota, while *glutamate synthase* (*NADPH*; *gltB*, and *gltD*) was higher in the big nodule microbiota. It was previously reported that bacteria or yeasts express distinct GDH isoenzymes for metabolic or biosynthetic processes. Based on nutrient type and availability, organisms that live in an amino acid rich environment use NAD^+^ − dependent GDH for their catabolic needs. In contrast, organisms utilizing inorganic N_2_ (nitrates or ammonia) use the NADP^+^ − specific GDH for their synthetic needs (Plaitakis et al., 2017) indicating that microbial communities in big and small nodules might use different GDH subunits for their catabolic or synthetic needs.

Like the N cycling process genes, also it was observed that most of the P cycling process genes were higher in abundance in the big nodules microbiota; however, *3-phytase* (E3.1.3.8; KO 1083), *4-phytase*/ *acid phosphatase* (*appA*; KO1093) and *phosphogluconate dehydrogenase* (*edd*; KO1690), *myo-inositol 2-dehydrogenase* (*iolG*), *6-phosphofructokinase 1* (*pfkA*), and *glucose-6-phosphate isomerase* (*GPI*) were higher in abundance in the small nodules microbiota. There might be some correlation between the presence of microbial communities and the abundance of phytases in the small nodules. Indeed, a recent report suggested that *Chitinophagales*, *Cytophagales*, *Sphingomonadales,* and *Xanthomonadales* were the order-level microbial indicators that positively correlated with the soil available phosphorus in agricultural soils (Wu et al., 2022). Most likely, this community of microbes capable of producing more phytases for hydrolyzing myo-inositol phosphates (phytates), which are abundant in many soil types, makes up a large portion of bioavailable soil P to improve plant growth and development (Balaban et al., 2017). Besides N- and P-related pathway genes, some other category of pathway genes were observed that specifically were enriched in the small nodules particularly in the late growth stages, suggesting that these pathway genes might play some role in the small nodules as well. The main objective of this study was the community profiling of two distinct field-grown nodule types in peanuts. Though the data could not fully support experimentally for biological or physiological functions for the predicted genes/subunits stated above, future studies will be required to answer this question.

## Conclusion

This research clearly observed that microbial inhabitants were quite different between the two types of nodules that formed on the same peanut roots under field conditions. The microbial community in the big nodules were predominantly colonized by the commercial rhizobial inoculum for the formation of N_2_-fixing nodules to fix atmospheric N_2_. In contrast, diverse native microbial communities co-inhabited with the commercial inoculum in the small nodules and dominated gradually during the plant growth and development. Based on our data analysis, the prediction of functional pathway of genes related to the N and P cycling processes as well as with the previous published reports indicated that the diverse microbial inhabitants in the small nodules might play some role in many ways (such as production of phytohormones, bioactive compounds, antibiotics, N and P availability, stress tolerance, phytoremediation for toxic chemicals etc.) for better peanut nodulation, growth, and development. Future studies with our recently isolated endophytes (un-published) from the same peanut nodules used for this microbiome study will certainly verify some of the above-mentioned functional roles and will provide clear data and recommendations for peanut improvement.

## Data availability statement

The data presented in the study are deposited in the https://www.ncbi.nlm.nih.gov/ repository, accession number PRJNA865795.

## Author contributions

MH designed and planned experimental procedures, collected samples for microbiome sequencing, performed all experimental works, bioinformatics, and statistical analysis, generated figures, and wrote the manuscript. PD designed the experimental field plot, collected and shipped plants to the laboratory from the field site, and revised the manuscript. TG provided advice for experimental design and plan, and edited and revised the manuscript. All authors contributed to the article and approved the submitted version.

## Conflict of interest

The authors declare that the research was conducted in the absence of any commercial or financial relationships that could be construed as a potential conflict of interest.

## Publisher’s note

All claims expressed in this article are solely those of the authors and do not necessarily represent those of their affiliated organizations, or those of the publisher, the editors and the reviewers. Any product that may be evaluated in this article, or claim that may be made by its manufacturer, is not guaranteed or endorsed by the publisher.

## Footnotes

^1^ https://qiime2.org

^2^ https://github.com/qiime2/q2-demux

^3^ https://github.com/qiime2/q2-phylogeny

^4^ https://github.com/qiime2/q2-diversity

## References

[ref1] Anderson M. J. (2001). A new method for non-parametric multivariate analysis of variance: NON-PARAMETRIC MANOVA FOR ECOLOGY. Austral Ecol. 26, 32–46. doi: 10.1111/j.1442-9993.2001.01070.pp.x

[ref2] Asaf S. Khan A. L. Khan M. A. Imran Q. M. Yun B.-W. Lee I.-J. (2017). Osmoprotective functions conferred to soybean plants via inoculation with Sphingomonas sp. LK11 and exogenous trehalose. Microbiol. Res. 205, 135–145. doi: 10.1016/j.micres.2017.08.009, PMID: 28942839

[ref3] Asaf S. Numan M. Khan A. L. Al-Harrasi A. (2020). *Sphingomonas*: from diversity and genomics to functional role in environmental remediation and plant growth. Crit. Rev. Biotechnol. 40, 138–152. doi: 10.1080/07388551.2019.1709793, PMID: 31906737

[ref4] Aserse A. A. Räsänen L. A. Aseffa F. Hailemariam A. Lindström K. (2013). Diversity of sporadic symbionts and nonsymbiotic endophytic bacteria isolated from nodules of woody, shrub, and food legumes in Ethiopia. Appl. Microbiol. Biotechnol. 97, 10117–10134. doi: 10.1007/s00253-013-5248-4, PMID: 24196581

[ref5] Bacon C. W. White J. F. (2016). Functions, mechanisms and regulation of endophytic and epiphytic microbial communities of plants. Symbiosis 68, 87–98. doi: 10.1007/s13199-015-0350-2

[ref6] Balaban N. P. Suleimanova A. D. Valeeva L. R. Chastukhina I. B. Rudakova N. L. Sharipova M. R.. (2017). Microbial Phytases and Phytate: exploring opportunities for sustainable phosphorus Management in Agriculture. AJMB. 07, 11–29. doi: 10.4236/ajmb.2017.71002

[ref7] Bender F. R. Alves L. C. da Silva J. F. M. Ribeiro R. A. Pauli G. Nogueira M. A.. (2022). Microbiome of nodules and roots of soybean and common bean: searching for differences associated with contrasting performances in symbiotic nitrogen fixation. IJMS. 23:12035. doi: 10.3390/ijms231912035, PMID: 36233333 PMC9570480

[ref8] Berg G. (2009). Plant–microbe interactions promoting plant growth and health: perspectives for controlled use of microorganisms in agriculture. Appl. Microbiol. Biotechnol. 84, 11–18. doi: 10.1007/s00253-009-2092-7, PMID: 19568745

[ref9] Bhattacharyya S. S. Furtak K. (2022). Soil–plant–microbe interactions determine soil biological fertility by altering Rhizospheric nutrient cycling and biocrust formation. Sustainability. 15:625. doi: 10.3390/su15010625

[ref10] Bogino P. Nievas F. Banchio E. Giordano W. (2011). Increased competitiveness and efficiency of biological nitrogen fixation in peanut via in-furrow inoculation of rhizobia. Eur. J. Soil Biol. 47, 188–193. doi: 10.1016/j.ejsobi.2011.01.005

[ref11] Bokulich N. A. Kaehler B. D. Rideout J. R. Dillon M. Bolyen E. Knight R.. (2018). Optimizing taxonomic classification of marker-gene amplicon sequences with QIIME 2’s q2-feature-classifier plugin. Microbiome. 6:90. doi: 10.1186/s40168-018-0470-z, PMID: 29773078 PMC5956843

[ref12] Bolyen E. Rideout J. R. Dillon M. R. Bokulich N. A. Abnet C. C. Al-Ghalith G. A.. (2019). Reproducible, interactive, scalable and extensible microbiome data science using QIIME 2. Nat. Biotechnol. 37, 852–857. doi: 10.1038/s41587-019-0209-9, PMID: 31341288 PMC7015180

[ref13] Boogerd F. C. van Rossum D. (1997). Nodulation of groundnut by *Bradyrhizobium*: a simple infection process by crack entry. FEMS Microbiol. Rev. 21, 5–27. doi: 10.1111/j.1574-6976.1997.tb00342.x

[ref14] Brader G. Compant S. Vescio K. Mitter B. Trognitz F. Ma L.-J.. (2017). Ecology and genomic insights into plant-pathogenic and plant-nonpathogenic endophytes. Annu. Rev. Phytopathol. 55, 61–83. doi: 10.1146/annurev-phyto-080516-035641, PMID: 28489497

[ref15] Callahan B. J. McMurdie P. J. Rosen M. J. Han A. W. Johnson A. J. A. Holmes S. P. (2016). DADA2: high-resolution sample inference from Illumina amplicon data. Nat. Methods 13, 581–583. doi: 10.1038/nmeth.3869, PMID: 27214047 PMC4927377

[ref16] Chen W.-M. Moulin L. Bontemps C. Vandamme P. Béna G. Boivin-Masson C. (2003). Legume symbiotic nitrogen fixation byβ-Proteobacteria is widespread inNature. J. Bacteriol. 185, 7266–7272. doi: 10.1128/JB.185.24.7266-7272.2003, PMID: 14645288 PMC296247

[ref17] Dazzo F. B. Gardiol A. E. (1984). “Host specificity in rhizobium-legume interactions” in Genes involved in microbe-plant interactions, plant gene research. eds. Verma D. P. S. Hohn T. (Vienna: Springer Vienna), 3–31.

[ref18] DeSantis T. Z. Hugenholtz P. Larsen N. Rojas M. Brodie E. L. Keller K.. (2006). Greengenes, a chimera-checked 16S rRNA gene database and workbench compatible with ARB. Appl. Environ. Microbiol. 72, 5069–5072. doi: 10.1128/AEM.03006-05, PMID: 16820507 PMC1489311

[ref19] Douglas G. M. Maffei V. J. Zaneveld J. R. Yurgel S. N. Brown J. R. Taylor C. M.. (2020). PICRUSt2 for prediction of metagenome functions. Nat. Biotechnol. 38, 685–688. doi: 10.1038/s41587-020-0548-6, PMID: 32483366 PMC7365738

[ref20] El-Akhal M. R. Rincón A. Arenal F. Lucas M. M. El Mourabit N. Barrijal S.. (2008). Genetic diversity and symbiotic efficiency of rhizobial isolates obtained from nodules of Arachis hypogaea in northwestern Morocco. Soil Biol. Biochem. 40, 2911–2914. doi: 10.1016/j.soilbio.2008.08.005

[ref21] Ellman-Stortz L. M. (2021). Influence of cover crop selection on soil microbial activity during a transition to organic agriculture. Doctoral Dissertation. Texas A&M University.

[ref22] Estaki M. Jiang L. Bokulich N. A. McDonald D. González A. Kosciolek T.. (2020). QIIME 2 enables comprehensive end-to-end analysis of diverse microbiome data and comparative studies with publicly available data. Curr. Protoc. Bioinformatics 70:e100. doi: 10.1002/cpbi.100, PMID: 32343490 PMC9285460

[ref23] Fadiji A. E. Ayangbenro A. S. Babalola O. O. (2020). Metagenomic profiling of the community structure, diversity, and nutrient pathways of bacterial endophytes in maize plant. Antonie Van Leeuwenhoek 113, 1559–1571. doi: 10.1007/s10482-020-01463-w, PMID: 32803452

[ref24] Fierer N. Jackson J. A. Vilgalys R. Jackson R. B. (2005). Assessment of soil microbial community structure by use of taxon-specific quantitative PCR assays. Appl. Environ. Microbiol. 71, 4117–4120. doi: 10.1128/AEM.71.7.4117-4120.2005, PMID: 16000830 PMC1169028

[ref25] Gan H. M. Buckley L. Szegedi E. Hudson A. O. Savka M. A. (2009). Identification of an *rsh* gene from a *Novosphingobium* sp. necessary for quorum-sensing signal accumulation. J. Bacteriol. 191, 2551–2560. doi: 10.1128/JB.01692-08, PMID: 19201802 PMC2668395

[ref26] Hardarson G. Golbs M. Danso S. (1989). Nitrogen fixation in soybean (Glycine max L. Merrill) as affected by nodulation patterns. Soil Biol. Biochem. 21, 783–787. doi: 10.1016/0038-0717(89)90171-5

[ref27] Harman G. E. Uphoff N. (2019). Symbiotic root-endophytic soil microbes improve crop productivity and provide environmental benefits. Scientifica. 2019, 1–25. doi: 10.1155/2019/9106395, PMID: 31065398 PMC6466867

[ref28] Held M. Hossain M. S. Yokota K. Bonfante P. Stougaard J. Szczyglowski K. (2010). Common and not so common symbiotic entry. Trends Plant Sci. 15, 540–545. doi: 10.1016/j.tplants.2010.08.001 20829094

[ref29] Hossain M. S. Liao J. James E. K. Sato S. Tabata S. Jurkiewicz A.. (2012). *Lotus japonicus ARPC1* is required for Rhizobial infection. Plant Physiol. 160, 917–928. doi: 10.1104/pp.112.202572, PMID: 22864583 PMC3461565

[ref30] Ji Z. J. Yan H. Cui Q. G. Wang E. T. Chen W. F. Chen W. X. (2017). Competition between rhizobia under different environmental conditions affects the nodulation of a legume. Syst. Appl. Microbiol. 40, 114–119. doi: 10.1016/j.syapm.2016.12.003, PMID: 28063627

[ref31] Khan A. L. Waqas M. Kang S.-M. Al-Harrasi A. Hussain J. Al-Rawahi A.. (2014). Bacterial endophyte Sphingomonas sp. LK11 produces gibberellins and IAA and promotes tomato plant growth. J. Microbiol. 52, 689–695. doi: 10.1007/s12275-014-4002-7, PMID: 24994010

[ref32] King C. A. Purcell L. C. (2001). Soybean nodule size and relationship to nitrogen fixation response to water deficit. Crop Sci. 41, 1099–1107. doi: 10.2135/cropsci2001.4141099x

[ref33] Kruskal W. H. Wallis W. A. (1952). Use of ranks in one-criterion variance analysis. J. Am. Stat. Assoc. 47, 583–621. doi: 10.1080/01621459.1952.10483441

[ref34] Kumar V. Kumar M. Sharma S. Prasad R., eds. (2017). Probiotics and plant health. Singapore: Springer Singapore.

[ref35] Lace B. Ott T. (2018). Commonalities and differences in controlling multipartite intracellular infections of legume roots by symbiotic microbes. Plant Cell Physiol. 59, 666–677. doi: 10.1093/pcp/pcy043, PMID: 29474692

[ref36] Li Y. Adams J. Shi Y. Wang H. He J.-S. Chu H. (2017). Distinct soil microbial communities in habitats of differing soil water balance on the Tibetan plateau. Sci. Rep. 7:46407. doi: 10.1038/srep46407, PMID: 28401921 PMC5388882

[ref37] Liu C.-W. Murray J. (2016). The role of flavonoids in nodulation host-range specificity: an update. Plan. Theory 5:33. doi: 10.3390/plants5030033, PMID: 27529286 PMC5039741

[ref38] Mandal S. Van Treuren W. White R. A. Eggesbø M. Knight R. Peddada S. D. (2015). Analysis of composition of microbiomes: a novel method for studying microbial composition. Microb. Ecol. Health. Dis. 26:27663. doi: 10.3402/mehd.v26.27663, PMID: 26028277 PMC4450248

[ref39] Martínez-Hidalgo P. Hirsch A. M. (2017). The nodule microbiome: N _2_ -fixing rhizobia do not live alone. Phytobiomes Journal. 1, 70–82. doi: 10.1094/PBIOMES-12-16-0019-RVW

[ref40] Mayhood P. Mirza B. S. (2021). Soybean root nodule and rhizosphere microbiome: distribution of Rhizobial and Nonrhizobial endophytes ed. Hideaki Nojiri. Appl Environ Microbiol. 87, e02884–e02820. doi: 10.1128/AEM.02884-20 PMC8117765 33674438

[ref41] McKee L. S. Martínez-Abad A. Ruthes A. C. Vilaplana F. Brumer H. (2019). Focused metabolism of β-glucans by the soil *Bacteroidetes* species Chitinophaga pinensis ed. Claire Vieille. Appl Environ Microbiol. 85, e02231–e02218. doi: 10.1128/AEM.02231-18 PMC6328771 30413479

[ref42] McMurdie P. J. Holmes S. (2013). Phyloseq: an R package for reproducible interactive analysis and graphics of microbiome census data ed. Michael Watson. PLoS One 8:e61217. doi: 10.1371/journal.pone.0061217, PMID: 23630581 PMC3632530

[ref43] Mendoza-Suárez M. Andersen S. U. Poole P. S. Sánchez-Cañizares C. (2021). Competition, nodule occupancy, and persistence of inoculant strains: key factors in the rhizobium-legume symbioses. Front. Plant Sci. 12:690567. doi: 10.3389/fpls.2021.690567, PMID: 34489993 PMC8416774

[ref44] Menéndez E. Robledo M. Jiménez-Zurdo J. I. Velázquez E. Rivas R. Murray J. D.. (2019). Legumes display common and host-specific responses to the rhizobial cellulase CelC2 during primary symbiotic infection. Sci. Rep. 9:13907. doi: 10.1038/s41598-019-50337-3, PMID: 31554862 PMC6761101

[ref45] Mongad D. S. Chavan N. S. Narwade N. P. Dixit K. Shouche Y. S. Dhotre D. P. (2021). MicFunPred: a conserved approach to predict functional profiles from 16S rRNA gene sequence data. Genomics 113, 3635–3643. doi: 10.1016/j.ygeno.2021.08.016, PMID: 34450292

[ref46] Moulin L. Munive A. Dreyfus B. Boivin-Masson C. (2001). Nodulation of legumes by members of the β-subclass of Proteobacteria. Nature 411, 948–950. doi: 10.1038/35082070, PMID: 11418858

[ref47] Msimbira L. A. Smith D. L. (2020). The roles of plant growth promoting microbes in enhancing plant tolerance to acidity and alkalinity stresses. Front. Sustain. Food Syst. 4:106. doi: 10.3389/fsufs.2020.00106

[ref48] Narayan N. R. Weinmaier T. Laserna-Mendieta E. J. Claesson M. J. Shanahan F. Dabbagh K.. (2020). Piphillin predicts metagenomic composition and dynamics from DADA2-corrected 16S rDNA sequences. BMC Genomics 21:56. doi: 10.1186/s12864-019-6427-1, PMID: 31952477 PMC6967091

[ref49] Nievas F. Bogino P. Nocelli N. Giordano W. (2012). Genotypic analysis of isolated peanut-nodulating rhizobial strains reveals differences among populations obtained from soils with different cropping histories. Appl. Soil Ecol. 53, 74–82. doi: 10.1016/j.apsoil.2011.11.010

[ref50] Olanrewaju O. S. Babalola O. O. (2019). Streptomyces: implications and interactions in plant growth promotion. Appl. Microbiol. Biotechnol. 103, 1179–1188. doi: 10.1007/s00253-018-09577-y, PMID: 30594952 PMC6394478

[ref51] Oldroyd G. E. D. (2013). Speak, friend, and enter: signalling systems that promote beneficial symbiotic associations in plants. Nat. Rev. Microbiol. 11, 252–263. doi: 10.1038/nrmicro2990, PMID: 23493145

[ref1003] Plaitakis A. Kalef-Ezra E. Kotzamani D. Zaganas I. Spanaki C. (2017). The Glutamate Dehydrogenase Pathway and Its Roles in Cell and Tissue Biology in Health and Disease. Biol. 6:11. doi: 10.3390/biology6010011 PMC5372004 28208702

[ref52] Poly F. Ranjard L. Nazaret S. Gourbière F. Monrozier L. J. (2001). Comparison of *nifH* gene pools in soils and soil microenvironments with contrasting properties. Appl. Environ. Microbiol. 67, 2255–2262. doi: 10.1128/AEM.67.5.2255-2262.2001, PMID: 11319109 PMC92864

[ref53] Poole P. Ramachandran V. Terpolilli J. (2018). Rhizobia: from saprophytes to endosymbionts. Nat. Rev. Microbiol. 16, 291–303. doi: 10.1038/nrmicro.2017.171, PMID: 29379215

[ref54] Pramanik K. Das A. Banerjee J. Das A. Chatterjee S. Sharma R.. (2020). Metagenomic insights into Rhizospheric microbiome profiling in lentil cultivars unveils differential microbial nitrogen and phosphorus metabolism under Rice-fallow Ecology. IJMS. 21:8895. doi: 10.3390/ijms21238895, PMID: 33255324 PMC7727700

[ref55] Prasad P. (1999). The effect of heat stress on fruit-set and fruit yield of groundnut (Arachis hypogaea L.). San Francisco, CA: Public Library of Science.

[ref56] Pruesse E. Quast C. Knittel K. Fuchs B. M. Ludwig W. Peplies J.. (2007). SILVA: a comprehensive online resource for quality checked and aligned ribosomal RNA sequence data compatible with ARB. Nucleic Acids Res. 35, 7188–7196. doi: 10.1093/nar/gkm864, PMID: 17947321 PMC2175337

[ref57] Quast C. Pruesse E. Yilmaz P. Gerken J. Schweer T. Yarza P.. (2012). The SILVA ribosomal RNA gene database project: improved data processing and web-based tools. Nucleic Acids Res. 41, D590–D596. doi: 10.1093/nar/gks1219, PMID: 23193283 PMC3531112

[ref58] Quilbé J. Montiel J. Arrighi J.-F. Stougaard J. (2022). Molecular mechanisms of intercellular Rhizobial infection: novel findings of an ancient process. Front. Plant Sci. 13:922982. doi: 10.3389/fpls.2022.922982, PMID: 35812902 PMC9260380

[ref1001] R Core Team. (2013). R: A Language and Environment for Statistical Computing. R Foundation for Statistical Computing. Vienna. Available at: http://www.R-project.org/

[ref59] Rodriguez-Conde S. Molina L. González P. García-Puente A. Segura A. (2016). Degradation of phenanthrene by Novosphingobium sp. HS2a improved plant growth in PAHs-contaminated environments. Appl. Microbiol. Biotechnol. 100, 10627–10636. doi: 10.1007/s00253-016-7892-y, PMID: 27722914

[ref1002] RStudio Team. (2020). RStudio: Integrated Development for R. RStudio, PBC, Boston, MA. Available at: http://www.rstudio.com/

[ref60] Rowland D. L. Smith C. Cook A. Mason A. Schreffler A. Bennett J. (2015). Visualization of Peanut nodules and seasonal nodulation pattern in different tillage systems using a Minirhizotron system. Peanut Science. 42, 1–10. doi: 10.3146/0095-3679-42.1.1

[ref61] Sarita S. Sharma P. K. Priefer U. B. Prell J. (2005). Direct amplification of rhizobial nodC sequences from soil total DNA and comparison to nodC diversity of root nodule isolates. FEMS Microbiol. Ecol. 54, 1–11. doi: 10.1016/j.femsec.2005.02.015, PMID: 16329967

[ref62] Segura A. Udaondo Z. Molina L. (2021). PahT regulates carbon fluxes in *Novosphingobium* sp. HR1a and influences its survival in soil and rhizospheres. Environ. Microbiol. 23, 2969–2991. doi: 10.1111/1462-2920.15509, PMID: 33817928 PMC8360164

[ref63] Sharma V. Bhattacharyya S. Kumar R. Kumar A. Ibañez F. Wang J.. (2020). Molecular basis of root nodule Symbiosis between Bradyrhizobium and ‘crack-entry’ legume groundnut (Arachis hypogaea L.). Plan. Theory 9:276. doi: 10.3390/plants9020276, PMID: 32093403 PMC7076665

[ref64] Swarnalakshmi K. Yadav V. Tyagi D. Dhar D. W. Kannepalli A. Kumar S. (2020). Significance of plant growth promoting Rhizobacteria in grain legumes: growth promotion and crop production. Plan. Theory 9:1596. doi: 10.3390/plants9111596, PMID: 33213067 PMC7698556

[ref65] Sy A. Giraud E. Jourand P. Garcia N. Willems A. de Lajudie P.. (2001). Methylotrophic *Methylobacterium* bacteria Nodulate and fix nitrogen in Symbiosis with legumes. J. Bacteriol. 183, 214–220. doi: 10.1128/JB.183.1.214-220.2001, PMID: 11114919 PMC94868

[ref66] Tajima R. Lee O. N. Abe J. Lux A. Morita S. (2007). Nitrogen-fixing activity of root nodules in relation to their size in Peanut (*Arachis hypogaea* L.). Plant Production Science. 10, 423–429. doi: 10.1626/pps.10.423

[ref67] Tatiana K.-W. Ibrahima N. Serge B. Benoit S. Philippe, de L. Marc N. (2003). Diversity of indigeneous bradyrhizobia associated with three cowpea cultivars (Vigna unguiculata (L.) Walp.) grown under limited and favorable water conditions in Senegal (West Africa). Afr. J. Biotechnol. 2, 13–22. doi: 10.5897/AJB2003.000-1003

[ref68] Taurian T. Ibañez F. Fabra A. Aguilar O. M. (2006). Genetic diversity of rhizobia Nodulating Arachis hypogaea L. in central Argentinean soils. Plant Soil 282, 41–52. doi: 10.1007/s11104-005-5314-5

[ref69] Vaidya P. Stinchcombe J. R. (2020). The potential for genotype-by-environment interactions to maintain genetic variation in a model legume–rhizobia mutualism. Plant Communications. 1:100114. doi: 10.1016/j.xplc.2020.100114, PMID: 33367267 PMC7747969

[ref70] Voisin A. S. (2003). Symbiotic N2 fixation activity in relation to C economy of Pisum sativum L. as a function of plant phenology. J. Exp. Bot. 54, 2733–2744. doi: 10.1093/jxb/erg290, PMID: 14563833

[ref71] Walker L. Lagunas B. Gifford M. L. (2020). Determinants of host range specificity in legume-rhizobia Symbiosis. Front. Microbiol. 11:585749. doi: 10.3389/fmicb.2020.585749, PMID: 33329456 PMC7728800

[ref72] Wemheuer F. Taylor J. A. Daniel R. Johnston E. Meinicke P. Thomas T.. (2020). Tax4Fun2: prediction of habitat-specific functional profiles and functional redundancy based on 16S rRNA gene sequences. Environmental Microbiome. 15:11. doi: 10.1186/s40793-020-00358-7, PMID: 33902725 PMC8067651

[ref73] Westhoek A. Clark L. J. Culbert M. Dalchau N. Griffiths M. Jorrin B.. (2021). Conditional sanctioning in a legume– *rhizobium* mutualism. Proc. Natl. Acad. Sci. U. S. A. 118:e2025760118. doi: 10.1073/pnas.2025760118, PMID: 33941672 PMC8126861

[ref74] Wu X. Rensing C. Han D. Xiao K.-Q. Dai Y. Tang Z.. (2022). Genome-resolved metagenomics reveals distinct phosphorus acquisition strategies between soil microbiomes ed. Nick Bouskill. mSystems. 7, e01107–e01121. doi: 10.1128/msystems.01107-21 PMC8751388 35014868

[ref75] Yang J. K. Zhou J. C. (2008). Diversity, phylogeny and host specificity of soybean and peanut bradyrhizobia. Biol. Fertil. Soils 44, 843–851. doi: 10.1007/s00374-008-0269-3

[ref76] Yilmaz P. Parfrey L. W. Yarza P. Gerken J. Pruesse E. Quast C.. (2014). The SILVA and “all-species living tree project (LTP)” taxonomic frameworks. Nucl. Acids Res. 42, D643–D648. doi: 10.1093/nar/gkt1209, PMID: 24293649 PMC3965112

[ref77] Zaiya Zazou A. Fonceka D. Fall S. Fabra A. Ibañez F. Pignoly S.. (2018). Genetic diversity and symbiotic efficiency of rhizobial strains isolated from nodules of peanut (Arachis hypogaea L.) in Senegal. Agric. Ecosyst. Environ. 265, 384–391. doi: 10.1016/j.agee.2018.06.001

[ref78] Zgadzaj R. Garrido-Oter R. Jensen D. B. Koprivova A. Schulze-Lefert P. Radutoiu S. (2016). Root nodule symbiosis in *Lotus japonicus* drives the establishment of distinctive rhizosphere, root, and nodule bacterial communities. Proc. Natl. Acad. Sci. U. S. A. 113, E7996–E8005. doi: 10.1073/pnas.1616564113, PMID: 27864511 PMC5150415

[ref79] Zipfel C. Oldroyd G. E. D. (2017). Plant signalling in symbiosis and immunity. Nature 543, 328–336. doi: 10.1038/nature22009 28300100

